# Development and application of survey-based artificial intelligence for clinical decision support in managing infectious diseases: A pilot study on a hospital in central Vietnam

**DOI:** 10.3389/fpubh.2022.1023098

**Published:** 2022-11-02

**Authors:** Kwanghyun Kim, Myung-ken Lee, Hyun Kyung Shin, Hyunglae Lee, Boram Kim, Sunjoo Kang

**Affiliations:** ^1^Department of Preventive Medicine, Yonsei University College of Medicine, Seoul, South Korea; ^2^Department of Public Health, Graduate School, Yonsei University, Seoul, South Korea; ^3^Graduate School of Public Health, Kosin University College of Medicine, Busan, South Korea; ^4^Acryl, Seoul, South Korea; ^5^FineHealthcare, Seoul, South Korea; ^6^Graduate School of Public Health, Yonsei University, Seoul, South Korea

**Keywords:** communicable diseases, artificial intelligence, Asia Southeastern, international health, low- & middle-income countries

## Abstract

**Introduction:**

In this study, we developed a simplified artificial intelligence to support the clinical decision-making of medical personnel in a resource-limited setting.

**Methods:**

We selected seven infectious disease categories that impose a heavy disease burden in the central Vietnam region: mosquito-borne disease, acute gastroenteritis, respiratory tract infection, pulmonary tuberculosis, sepsis, primary nervous system infection, and viral hepatitis. We developed a set of questionnaires to collect information on the current symptoms and history of patients suspected to have infectious diseases. We used data collected from 1,129 patients to develop and test a diagnostic model. We used XGBoost, LightGBM, and CatBoost algorithms to create artificial intelligence for clinical decision support. We used a 4-fold cross-validation method to validate the artificial intelligence model. After 4-fold cross-validation, we tested artificial intelligence models on a separate test dataset and estimated diagnostic accuracy for each model.

**Results:**

We recruited 1,129 patients for final analyses. Artificial intelligence developed by the CatBoost algorithm showed the best performance, with 87.61% accuracy and an F1-score of 87.71. The F1-score of the CatBoost model by disease entity ranged from 0.80 to 0.97. Diagnostic accuracy was the lowest for sepsis and the highest for central nervous system infection.

**Conclusion:**

Simplified artificial intelligence could be helpful in clinical decision support in settings with limited resources.

## Introduction

Although there have been successes in decreasing the disease burden of infectious diseases, there has been a dramatic emergence of infectious diseases, and they remain significant public health challenges (1). Southeast Asia is one of the ‘hot spots' for infectious diseases, and it has experienced a rapid surge of infectious diseases and emerging infectious diseases. This rapid increase in disease burden results from multiple reasons, including environmental factors (2, 3), changes in biodiversity (4), and economic factors (5). Several infectious diseases such as dengue fever (6), malaria (7), and central nervous system infection (8) are imposing a heavy disease burden on Southeast Asia as their prevalence grows.

Artificial intelligence (AI) is increasingly being used in fields of medical practices. Particularly, AI was proven to help assist medical decision-making. For example, AIs for diagnosing respiratory diseases (9, 10), cardiovascular diseases (11), and infectious diseases (12, 13) have been developed and used for diagnostic assistance. Particularly for infectious disease management, AIs to support clinical decision-making for managing emerging infectious diseases, including tuberculosis (14, 15), vector-borne diseases (16, 17) and COVID-19 (18, 19) had been developed. Such previous models have achieved high diagnostic accuracy, proving their effectiveness in medical decision-making for infectious diseases (16, 20, 21).

Previously, regression-based classifiers had been widely used for developing prediction model for clinical decision making, as it is highly intuitive and often one of the models with highest predictability for dichotomous outcome (22–24). However, in order to apply logistic regression model, data must conform statistical assumptions such as avoidance of multicollinearity of independent variables and independence of observation (25). Machine learning classifiers can avoid this issue and can be applied to wider range of unstructured dataset, and therefore have been implemented to develop and improve artificial models for disease (20, 26). A number of machine learning methods, including artificial neural networks (27), XGBoost (28), and support vector machine methods (29, 30) are being used for diagnostic assistance and clinical decision making.

Up to this date, however, little has been documented on applying AI for public health in resource-limited settings of low- and middle-income countries (LMICs) (31). Although there are vigorous activities on developing and using AI for public health in resource-limited settings (32–34), its application is still at its elementary level. Several difficulties in the application of AIs, such as challenges in building and maintenance of expert systems (35), limitations in IT infrastructure (36), differences in socioeconomic contexts (36, 37) and lack of personnel to supervise the procedure of development and application (38) hinder effective use of AIs for public health in the resource-limited setting of LMICs. To increase the availability and accessibility of AI in LMICs, AI needs to be tailored to the resource-limited settings and fit for local sociomedical contexts and infrastructure needs (31, 39).

Our objective of this research was to develop a simplified version of AI that we could effectively apply in resource-limited settings. We conducted a pilot study collaborating with local authorities and healthcare institutions in Da Nang, Vietnam. We tried to develop an AI for diagnosing infectious diseases that impose a heavy disease burden in the Central region of Vietnam.

## Materials and methods

### Developing questionnaire

We selected seven infectious disease categories that were most common in central Vietnam or imposed a heavy disease burden on central Vietnam: mosquito-borne diseases, acute gastroenteritis, respiratory tract infection including coronavirus disease (COVID-19), pulmonary tuberculosis, sepsis, central nervous system (CNS) infection, and viral hepatitis. Classification of disease entity was done following ICD-10 diagnostic codes (Supplemental material 1). We used the Delphi method to develop questionnaires for patient assessment and history taking. A preventive medicine specialist in English initially created a questionnaire. Then, it was reviewed by a Korean preventive medicine specialist, a Korean public health specialist, two Vietnamese internal medicine specialists, a Vietnamese public health specialist, and an English-Vietnamese interpreter. After review and feedback, a professional translator translated the questionnaire set into Vietnamese. We have attached the final questionnaire as Supplemental material 2. Before data collection, we conducted two rounds of a pilot study to receive feedback and edit questionnaires: once on 5 Vietnamese individuals residing in Korea and once on 10 Vietnamese recruited from Da Nang.

After the pilot study, a Korean preventive medicine specialist developed an instruction manual for research personnel, which was used to educate physicians and nurses at the Department of Tropical Medicine, Da Nang Hospital, before conducting the survey. In addition, a survey application was developed and installed to tablet PCs used for data collection.

### Study participants

We recruited patients who had been diagnosed with either of the target disease entities. About 160 participants for each disease category were recruited to ensure the model's predictive power. Due to decreased outpatient visits due to the COVID-19 outbreak in Vietnam, we used a two-track recruitment strategy. Patients admitted to the department of tropical medicine, Da Nang Hospital, Da Nang Hospital, and Da Nang Lung Hospital from November 8th, 2021, to January 1st, 2022, were recruited and responded to the survey. Simultaneously, research personnel conducted a phone survey on patients who had been diagnosed with target diseases. We recruited a total number of 1,131 patients either prospectively or retrospectively. We excluded two participants who were diagnosed with other conditions were excluded from the final analysis.

### Data collection and management

All participants then underwent an interview with a developed questionnaire and physical examination. Data was collected using the application on a tablet PC, then directly transmitted to the server. We measured systolic and diastolic blood pressure with a standardized sphygmomanometer after 5 min of rest. In addition, other vital signs such as pulse rate, respiratory rate, and body temperature were measured by physicians and nurses at the Department of Tropical Medicine, Da Nang Hospital. For retrospectively recruited participants, we collected records on electronic medical records (EMRs) of Da Nang Hospital, and we did an additional telephone survey to collect data. After the survey, the physician gave a final diagnosis to participants and was compiled with its corresponding ICD-10 codes. Following ICD-10 codes and diagnosis, participants were categorized into seven disease entity subgroups.

### Development of artificial intelligence (AI) for disease classification

Survey results were processed to a dataset with 211 independent variables. We used three different algorithms (XGBoost, LightGBM, CatBoost) to develop AI for prediction and compared the predictive accuracy of the three models. XGBoost, one of the Gradient Boosting Models (GBM), is an ensemble model of decision trees (40). By implementing parallel processing and CART (Classification and Regression Tree) model-based regression, XGBoost works extremely faster compared to the previous gradient models and efficiently handles overfitting problem of GBM (40). LightGBM utilizes gradient-based one-side sampling (GOSS) and Exclusive Feature Bundling (EFB), adopting the leaf-wise tree grwoth algorithm unlike level-wise growth algorithms commonly used in previous GBMs (41). LightGBM has considerably lower false predictions due to application of leaf-wise tree growth, and it has faster training speed and lesser memory usage compared to conventional GBMs such as XGBoost by efficiently reducing the number of data instances and features (41). However, due to such characteristic, insufficient sample size may cause overfitting in LightGBM. CatBoost builds the base model with the residual error of independently sampled sub-dataset. The model is continuously updated by taking the residual error with the remaining dataset, solving the problem of prediction-shift (42). In addition, CatBoost creates clusters for each category during training, efficiently reflecting the categorical features to the model algorithm (42). The implementation of ordered boosting algorithm and ordered target statistics (TS) accelerates the training process, increases predictability, and reduces the possibility of overfitting (42). Moreover, the base parameters are already optimized in CatBoost, which minimizes the need of hyperparameter tuning (42).

We pre-processed the raw dataset into numerical dataset. For numeric variables, we modified the value to “– 1” for missing values and did not modify other responses before including them into the final dataset. For non-numeric features, we modified the value to “1” if the answer exists and to “– 1” if not. We did not consider the specific response of the question, as the presence of the feature was more important in predicting results.

**Figure 1:**
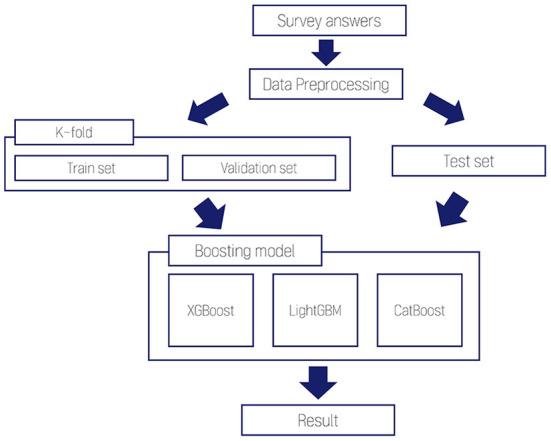
Architecture of artificial intelligence model construction.

The preprocessed dataset was divided into a training set, validation set, and test set. First, we divided the preprocessed data into a training set and a test set with a ratio of 9:1. Although there is no profound theoretical background on optimal training/test split ratio, a recent study had suggested that optimal training/test ratio with p features is approximately $\sqrt{p}$:1 (43). Since our study has 211 features, the optimal ratio would have been ~14.51:1, but we followed the precedent of previous studies on AI development with clinical purposes (44–46). Then we separated the training set into four groups for 4-fold cross-validation, rotationally using each set for validation and the rest for training (Figure 1). We conducted 9:1 splitting to secure the number of cases used for training and cross-validation. Finally, we modified the value of selected hyperparameters for each algorithm to optimize the model performance. The optimal value of each hyperparameter is shown in Supplemental material 3.

Feature importance, which is defined as ‘the increase in the model's prediction error after permuting the feature, was calculated for each variable included in the prediction model (47, 48). Global diagnostic accuracy and F1-score were measured to evaluate the developed prediction model. Global diagnostic accuracy was defined as the proportion of correct classification (**Equation 1**). Precision is defined as the proportion of true positive among samples classified as true (**Equation 2**). Recall, or sensitivity is defined as the proportion of true positive among positive samples (**Equation 3**). F1-score, which is a harmonic mean of precision and recall, shows the model performance of the developed model (**Equation 4**). After the global test for AI performance, we calculated performance parameters (precision, recall, specificity, and F1-score) by disease category.


(1) $$\begin{array}{lll} Accuracy & = & \;\frac{TP+TN}{TP+TN+FP+FN} \end{array}$$

(2) $$\begin{array}{lll} Precision & = & \;\frac{TP}{TP+FP} \end{array}$$

(3) $$\begin{array}{lll} Recall\;\left(Sensitivity\right) & = & \;\frac{TP}{TP+FN} \end{array}$$

(4) $$\begin{array}{lll} F1-score & = & \;2\;\times \frac{Precision\times \;Recall}{Precision+\;Recall} \end{array}$$

(5) $$\begin{array}{lll} Specificity & = & \;\frac{TN}{TN+FP} \end{array}$$

Multi-comparison table for each classifier was constructed for further comparison of model performance.

### Ethics approval

The institutional review board of Da Nang Hospital, Da Nang, Vietnam reviewed and approved the study protocol (Supplemental material 4). In addition, we acquired informed consent from all participants of this study. All procedures were contributing to this work to comply with the ethical standards of the relevant national and institutional committees on human experimentation and with the 1975 Declaration of Helsinki, which was revised in 2008.

## Results

### Participant characteristics

We recruited a total number of 1,129 participants for the survey. The number of patients diagnosed with mosquito-borne diseases, acute gastroenteritis, viral hepatitis, respiratory tract infection, pulmonary tuberculosis, sepsis, and CNS infection was 163 (14.44%), 162 (14.35%), 162 (14.35%), 158 (13.99%), 161 (14.26%), 161(14.26%) and 162 (14.44%), respectively. The mean age of participants was 45.96 years (standard deviation [SD] 17.88) at the point of diagnosis. Six hundred sixty-nine patients (59.26%) were men, and 460 (40.74%) were female.

There were significant differences in patterns of symptoms by disease entity. In mosquito-borne disease, fever/chill, fatigue, headache, anorexia, diarrhea, and myalgia were common. Gastrointestinal tract symptoms such as diarrhea, constipation, and abdominal pain were common in acute gastroenteritis. Systematic symptoms such as fever/chill and fatigue were the most common in sepsis and viral hepatitis, with no other prominent symptoms present (Table 1).

**Table 1:** Characteristics of study participants by disease entity.

	**1. Mosquito-borne disease (*N* = 163)**	**2. Acute infectious gastroenteritis (*N* = 162)**	**3. Respiratory tract infection (*N* = 158)**	**4. Pulmonary tuberculosis (*N* = 161)**	**5. Sepsis (*N* = 161)**	**6. CNS infection (*N* = 162)**	**7. Viral hepatitis (*N* = 162)**	**Total (*N* = 1,129)**
Age, mean (SD)	36.12 (14.73)	45.8 (19.9)	48.86 (19.13)	46.40 (14.85)	56.02 (16.47)	44.05 (16.99)	44.46 (16.25)	45.96 (17.88)
Sex, N (%)								
Male	92 (56.44)	70 (43.21)	77 (48.73)	123 (76.40)	95 (59.01)	100 (61.73)	112 (69.14)	669 (59.26)
Female	71 (45.56)	92 (56.79)	81 (51.27)	38 (23.60)	66 (40.99)	62 (0.13)	50 (30.86)	461 (40.74)
Height, mean (SD)	162.01 (10.65)	159.78 (9.17)	159.99 (8.05)	162.81 (6.31)	159.59 (7.50)	162.20 (7.23)	163.04 (6.91)	161.36 (8.21)
Body weight, mean (SD)	58.38 (9.73)	56.87 (11.05)	56.96 (10.42)	52.78 (9.32)	55.73 (8.94)	56.76 (8.8)	57.24 (8.91)	56.38 (9.76)
BMI, mean (SD)	22.7 (9.13)	22.17 (3.18)	22.18 (3.3)	19.73 (3.37)	21.83 (2.84)	21.52 (2.64)	21.48 (2.62)	21.65 (4.53)
Waist circumference, mean (SD)	78.11 (10.61)	73.15 (9.39)	72.2 (8.99)	73.14 (10.23)	74.18 (11.73)	77.23 (9.74)	77.65 (11.41)	75.1 (10.60)
Systolic blood pressure, mean (SD)	113.68 (10.27)	117.72 (15.84)	119.81 (15.28)	116.70 (14.27)	113.51 (19.13)	118.81 (13.37)	116.53 (11.97)	116.67 (14.69)
Diastolic blood pressure, mean (SD)	70 (9.07)	71.6 (9.42)	73.23 (8.73)	71.77 (9.4)	69.38 (11.83)	73.26 (8.59)	70.56 (7.72)	71.41 (9.43)
Pulse rate, mean (SD)	80.85 (5.72)	81.02 (9.83)	87.63 (13.7)	83.81 (8.77)	88.85 (12.25)	84.36 (8.92)	79.09 (5.83)	83.65 (10.23)
Respiratory rate, mean (SD)	19.99 (0.73)	20.35 (3.24)	19.58 (1.83)	20.53 (1.14)	21.42 (5.73)	20.73 (4.01)	20.69 (3.41)	20.47 (3.34)
Current symptoms, N (%)	0(0)	0(0)	0(0)	0 (0)	0 (0)	0 (0)	0 (0)	0 (0)
Systematic								
General weakness	14 (8.59)	3 (1.85)	21 (13.29)	61 (37.89)	27 (16.77)	26 (16.15)	10 (6.17)	162 (14.35)
Fever	159 (97.55)	65 (40.12)	56 (35.44)	77 (47.83)	135 (83.85)	152 (93.83)	8 (4.94)	652 (57.75)
Chill	126 (77.3)	30 (18.52)	31 (19.62)	61 (37.89)	76 (47.2)	122 (75.31)	1 (0.62)	447 (39.59)
Fatigue	158 (96.93)	83 (51.23)	96 (60.76)	109 (67.7)	141 (87.58)	152 (93.83)	128 (79.01)	867 (76.79)
Loss of appetite	43 (26.38)	7 (4.32)	45 (28.48)	6 (3.73)	41 (25.47)	19 (11.73)	13 (8.02)	174 (15.41)
Change in body weight	12 (7.36)	4 (2.47)	8 (5.06)	87 (54.04)	14 (8.70)	9 (5.56)	22 (13.58)	156 (13.82)
Pain	59 (36.19)	1 (0.62)	3 (1.90)	5 (3.11)	10 (6.21)	10 (6.17)	1 (0.62)	89 (7.88)
HEENT								
Headache	156 (95.71)	29 (17.90)	23 (14.56)	9 (5.59)	36 (22.36)	154 (95.06)	3 (1.85)	410 (36.32)
Neck stiffness	1 (0.61)	0 (0)	0 (0)	1 (0.62)	3 (1.86)	87 (53.70)	0 (0)	92 (8.15)
Dizziness	48 (29.45)	11 (6.79)	10 (6.33)	4 (2.48)	11 (6.83)	70 (43.21)	3 (1.85)	157 (13.91)
Vertigo	47 (28.83)	16 (9.88)	11 (6.96)	5 (3.11)	18 (11.18)	40 (24.69)	5 (3.09)	142 (12.58)
Sore throat	14 (8.59)	2 (1.23)	42 (26.58)	6 (3.73)	2 (1.24)	4 (2.47)	0 (0)	70 (6.20)
Rhinorrhea	3 (1.84)	3 (1.85)	16 (10.13)	1 (0.62)	0 (0)	5 (3.09)	0 (0)	28 (2.48)
Nasal stiffness	3 (1.84)	2 (1.23)	31 (19.62)	5 (3.11)	1 (0.62)	2 (1.23)	0 (0)	44 (3.90)
Nasal bleeding	2 (1.23)	0 (0)	0 (0)	0 (0)	1 (0.62)	2 (1.23)	0 (0)	5 (0.44)
Respiratory								
Cough	22 (13.5)	10 (6.17)	105 (66.46)	135 (83.85)	29(18.01)	12 (7.41)	2 (1.23)	315 (27.90)
Sneeze	6 (3.68)	1 (0.62)	11 (6.96)	1 (0.62)	1(0.62)	1 (0.62)	0 (0)	21 (1.86)
Sputum	4 (2.45)	2 (1.23)	44 (27.85)	91 (56.52)	17(10.56)	4 (2.47)	0 (0)	162 (14.35)
Hemoptysis	0 (0)	0 (0)	5 (3.16)	35 (21.74)	1 (0.62)	0 (0)	0 (0)	41 (3.63)
Dyspnea	8 (4.91)	12 (7.41)	34 (21.52)	58 (36.02)	39 (24.22)	5 (3.09)	6 (3.70)	162 (14.35)
Dyspnea on exertion	2 (1.23)	4 (2.47)	10 (6.33)	56 (34.78)	23 (14.29)	3 (1.85)	6 (3.70)	104 (9.21)
Orthopnea	4 (2.45)	4 (2.47)	10 (6.33)	40 (24.84)	23 (14.29)	4 (2.47)	6 (3.70)	91 (8.06)
Cardiovascular								
Chest discomfort or pain	8 (4.91)	11 (6.79)	14 (8.86)	62 (38.51)	13 (8.07)	0 (0)	9 (5.56)	117 (10.36)
Radiating pain	2 (1.23)	0 (0)	3 (1.9)	7 (4.35)	1 (0.62)	0 (0)	0(0)	13 (1.15)
Palpitation	6 (3.68)	2 (1.23)	0 (0)	1 (0.62)	2 (1.24)	0 (0)	1 (0.62)	12 (1.06)
Coldness in limbs	15 (9.20)	1 (0.62)	3 (1.90)	1 (0.62)	2 (1.24)	0 (0)	0(0)	22 (1.95)
Cyanosis	4 (2.45)	0 (0)	5 (3.16)	0 (0)	1 (0.62)	0 (0)	0(0)	10 (0.89)
Gastrointestinal								
Anorexia	119 (73.01)	23 (14.2)	36 (22.78)	75 (46.58)	75 (46.58)	127 (78.40)	112 (69.14)	567 (50.22)
Nausea	96 (58.90)	109 (67.28)	14 (8.86)	4 (2.48)	62 (38.51)	112 (73.46)	28 (17.28)	432 (38.26)
Vomiting	34 (20.86)	94 (58.02)	7 (4.43)	1 (0.62)	39 (24.22)	92 (56.79)	12 (3.91)	279 (24.71)
Diarrhea	32 (19.63)	150 (92.59)	5 (3.16)	6 (3.73)	46 (28.57)	5 (3.09)	10 (6.17)	254 (22.50)
Constipation	1 (0.61)	3 (1.85)	2 (1.27)	2 (1.24)	6 (3.73)	35 (21.60)	5 (3.09)	54 (4.78)
Abdominal pain	31 (19.02)	158 (97.53)	12 (7.59)	8 (4.97)	70 (43.48)	16 (9.87)	61 (37.65)	356 (31.53)
Hematemesis	1 (0.61)	1 (0.62)	0 (0)	1 (0.62)	1 (0.62)	0 (0)	3 (1.85)	7 (0.62)
Melena	2 (1.23)	1 (0.62)	0 (0)	2 (1.24)	3 (1.86)	0 (0)	4 (2.47)	12 (1.06)
Hematochezia	0 (0)	1 (0.62)	0 (0)	0 (0)	1 (0.62)	0 (0)	0 (0)	2 (0.18)
Jaundice	1 (0.61)	1 (0.62)	0 (0)	0 (0)	10 (6.21)	0 (0)	59 (36.42)	71 (6.29)
Ascites	0 (0)	0 (0)	0 (0)	0 (0)	7 (4.35)	0 (0)	13 (8.02)	20 (1.77)
Genitourinary								
Dysuria	2 (1.23)	0 (0)	5 (3.16)	3 (1.86)	11 (6.83)	7 (4.35)	1 (0.62)	29 (2.57)
Gross hematuria	0 (0)	0 (0)	0 (0)	1 (0.62)	4 (2.48)	0 (0)	0 (0)	5 (0.44)
Urgency	1 (0.61)	0 (0)	0 (0)	2 (1.24)	6 (3.73)	10 (6.17)	1 (0.62)	20 (1.77)
Frequency	6 (3.68)	3 (1.85)	1 (0.63)	3 (1.86)	10 (6.21)	3 (1.85)	1 (0.62)	27 (2.39)
Hesitancy	0 (0)	0 (0)	1 (0.63)	0 (0)	2 (1.24)	18(1.43)	0 (0)	21 (1.86)
Flank pain	8 (4.91)	1 (0.62)	0 (0)	4 (2.48)	15 (9.32)	2(0.16)	1 (0.62)	31 (2.75)
Edema	0 (0)	5 (3.09)	1 (0.63)	2 (1.24)	7 (4.35)	0 (0)	11 (6.79)	26 (2.30)
Skin & musculoskeletal	145 (88.96)	14 (8.64)	45 (28.48)	15 (9.32)	48 (29.81)	13(8.02)	0 (0)	280 (24.80)
Myalgia	66 (40.49)	6 (3.7)	26 (16.46)	6 (3.73)	17 (10.56)	9 (5.56)	0 (0)	131(11.58)
Joint pain	11 (6.75)	8 (4.94)	16 (10.13)	8 (4.97)	18 (11.18)	3 (1.85)	0 (0)	65(5.75)
Joint stiffness	0 (0)	0 (0)	2 (1.27)	0 (0)	4 (2.48)	0 (0)	0 (0)	6(0.53)
Rash	30 (18.4)	0 (0)	0 (0)	1 (0.62)	2 (1.24)	1 (0.62)	0 (0)	34(3.01)
Heat	38 (23.31)	0 (0)	1 (0.63)	0 (0)	7 (4.35)	0 (0)	0 (0)	47(4.16)
Neurologic								
Altered consciousness	1 (0.61)	2 (1.23)	4 (2.53)	0 (0)	31 (19.25)	78 (48.15)	0 (0)	117(10.34)
Altered orientation	1 (0.61)	0 (0)	2 (1.27)	1 (0.62)	19 (11.8)	69 (42.59)	0 (0)	92(8.13)
Altered cognition	1 (0.61)	1 (0.62)	3 (1.90)	0 (0)	27 (16.77)	68 (41.98)	0 (0)	100(8.84)
Altered sensory function	2 (1.23)	0 (0)	2 (1.27)	1 (0.62)	2 (1.24)	8 (4.94)	0 (0)	15(1.33)
Altered motor function	3 (1.84)	4 (2.47)	7 (4.43)	2 (1.24)	13 (8.07)	19 (11.73)	0 (0)	48(4.24)
Tremor	19 (11.66)	2 (1.23)	3 (1.90)	1 (0.62)	4 (2.48)	7 (4.32)	0 (0)	36(3.18)
Spasm	1 (0.61)	0 (0)	1 (0.63)	0 (0)	1 (0.62)	25 (15.43)	0 (0)	28(2.48)
Plegia	0 (0)	0 (0)	1 (0.63)	0 (0)	2 (1.24)	15 (9.26)	0 (0)	18(1.59)
Paresis	0 (0)	1 (0.62)	0 (0)	0 (0)	0 (0)	10 (6.17)	0 (0)	11(0.97)
Gait disturbance	2 (1.23)	2 (1.23)	4 (2.53)	0 (0)	4 (2.48)	12 (7.41)	0 (0)	24(2.12)
Aphasia	0 (0)	0 (0)	2 (1.27)	0 (0)	3 (1.86)	4 (2.47)	0 (0)	9(0.8)

### Performance comparison of developed artificial intelligence

**Figure 2:**
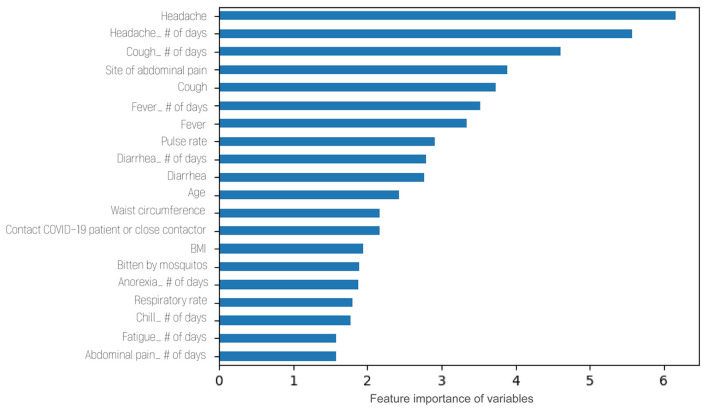
Feature importance of variables included in artificial intelligence model.

Figure 2 presents the top 20 influential features of the global model with feature importance values. The X-axis indicates the relative feature importance, and the y-axis represents the names of the feature. Headache and its duration were the most influential variables, followed by cough, fever, and pulse rate. Feature importance of variables by disease entity is presented in Supplemental material 5.

Table 2 shows the global diagnostic accuracy and F1-score of developed AI models. The model developed by the CatBoost algorithm showed the highest global diagnostic accuracy of 87.61%, and the model developed by the XGBoost algorithm showed the lowest global accuracy of 83.18%. We conducted 4-fold cross-validation on the model developed by the CatBoost algorithm, the model with the highest global diagnostic accuracy. Results from 4-fold cross validation also showed that diagnostic accuracy was the highest in CatBoost classifier: mean diagnostic accuracy of XGBoost, LightGBM, and CatBoost classifier were 0.832 (standard deviation [SD] 0.019), 0.839 (SD 0.009), and 0.850 (SD 0.019), respectively. Mean F1-score of XGBoost, LightGBM, and CatBoost classifier were 0.833 (SD 0.018), 0.840 (SD 0.009), and 0.850 (SD 0.019), respectively.

**Table 2:** Diagnostic accuracy and F1-score of AI models developed under different algorithm.

	**Precision**	**Recall**	**Specificity**	**F1-score**
XGBoost: Global accuracy = 83.18%, Global F1-score = 83.26
Mosquito-borne disease	0.81	0.76	0.96	0.79
Acute gastroenteritis	0.87	0.81	0.97	0.84
Respiratory tract infection including COVID-19	0.86	0.75	0.97	0.80
Pulmonary tuberculosis	0.83	0.94	0.96	0.88
Sepsis	0.70	0.88	0.93	0.78
Central nervous system infection	0.93	0.81	0.98	0.87
Viral hepatitis	0.88	0.88	0.97	0.88
LightGBM: Global accuracy = 82.74%, Global F1-score = 83.33
Mosquito-borne disease	0.81	0.79	0.96	0.79
Acute gastroenteritis	0.87	0.81	0.97	0.84
Respiratory tract infection including COVID-19	0.87	0.81	0.97	0.84
Pulmonary tuberculosis	0.88	0.88	0.97	0.88
Sepsis	0.68	0.81	0.93	0.74
Central nervous system infection	0.88	0.88	0.97	0.88
Viral hepatitis	0.88	0.88	0.97	0.88
CatBoost: Global accuracy = 87.61%, Global F1-score = 87.71
Mosquito-borne disease	0.88	0.88	0.97	0.88
Acute gastroenteritis	0.93	0.88	0.98	0.90
Respiratory tract infection including COVID-19	0.87	0.81	0.97	0.84
Pulmonary tuberculosis	0.87	0.81	0.97	0.84
Sepsis	0.74	0.88	0.94	0.80
Central nervous system infection	1.00	0.94	1.00	0.97
Viral hepatitis	0.88	0.94	0.97	0.91

Multi-comparison table for each classifier showed that performance of CatBoost algorithm was relatively higher compared to XGBoost and LightGBM. False prediction rates of XGBoost, LightGBM and CatBoost classifier were 16.81, 16.81, and 11.76, respectively. (Table 3) The result from multi-comparison table is concurrent with Table 2, which shows higher global accuracy in CatBoost classifier compared to other two classifiers.

**Table 3:** Results from 4-fold cross validation on the model developed by XGBoost, LightGBM, and CatBoost algorithm.

	**Accuracy**	**F1-score**
XGBoost	0.832 (±0.019)	0.833 (±0.018)
LightGBM	0.839 (±0.009)	0.840 (±0.009)
CatBoost	0.850 (±0.019)	0.850 (±0.019)

Parameters on AI performance by disease category are shown below in Table 4. Precision and recall were the lowest in the “sepsis” category and the highest in the “CNS infection” category. On the other hand, precision, recall, and sensitivity were generally higher in AI developed by the CatBoost algorithm, with exceptions such as the “mosquito-borne disease” category and the “CNS infection” category.

**Table 4:** Multi-class confusion matrix.

**XGBoost**	**Predicted class**						
	**1**	**2**	**3**	**4**	**5**	**6**	**7**
1	13	0	1	2	1	0	0
2	0	13	1	0	1	0	1
3	1	0	12	0	2	0	1
4	0	1	0	15	0	0	0
5	1	0	0	0	14	1	0
6	0	1	0	0	2	13	0
7	1	0	0	1	0	0	14
**LightGBM**	**Predicted class**						
	**1**	**2**	**3**	**4**	**5**	**6**	**7**
1	**13**	0	1	1	2	0	0
2	0	**13**	0	0	2	0	1
3	1	0	**13**	1	0	1	0
4	0	1	0	**14**	1	0	0
5	2	1	0	0	**13**	0	0
6	0	0	1	0	0	**14**	1
7	0	0	0	0	1	1	**14**
**CatBoost**	**Predicted class**						
	**1**	**2**	**3**	**4**	**5**	**6**	**7**
1	**15**	0	1	0	1	0	0
2	0	**14**	1	1	0	0	0
3	1	0	**13**	1	1	0	0
4	0	0	0	**13**	3	0	0
5	0	1	0	0	**14**	0	1
6	0	0	0	0	0	**15**	1
7	1	0	0	0	0	0	**15**

## Discussion

In this pilot study, we achieved around 85% of global diagnostic accuracy with AI developed with limited data, only including medical histories, physical examination results, and symptom assessments. The global accuracy increases up to 90% after excluding sepsis, which is a condition that requires a complex diagnostic procedure for accurate assessment (49). The accuracy we have achieved in this study is reasonably high, considering that the prediction model developed in this study did not include any laboratory test results or radiologic findings. For instance, machine learning algorithms for predicting hepatitis C in patients enrolled in National Treatment Program of HCV patients in Egypt showed accuracy of 66–84.4%, while our CatBoost model showed accuracy of 88% (50). Accuracy of predicting pulmonary tuberculosis was 87% in our model developed by CatBoost algorithm, while previous models based on chest X-ray showed area under curve of 0.75–0.99 (51). Considering that artificial intelligence in this study relied solely on survey questionnaire, diagnostic accuracy of the models developed in this study is relatively high compared to previous studies.

The global diagnostic accuracy of AI developed in this study is relatively low compared to other AIs, usually achieving 90% of higher diagnostic accuracy (12, 52). However, these AIs typically rely on additional tests such as radiologic studies, laboratory tests, and pathologic results. Our study developed a survey-based AI without additional tests except for vital sign assessments and physical examination, making it applicable to resource-limited settings (53).

While the developed AI cannot be a replacement for the clinical decision-making process, we could use it for screening tests and initial disease evaluation under circumstances of insufficient medical expertise, which is a common condition in LMICs (53, 54). However, there are significant challenges in applying AI to LMICs, mostly from local governance capacity and AI literacy (31, 55). Using tailored AI with high cost-efficacy and collaboration with experts in AI development and management will effectively screen and manage the disease of interest. Our study showed that simplified survey-based AI provides certain benefits in detecting and controlling infectious diseases in the Central region of Vietnam. We are planning to improve the diagnostic accuracy of AI further and evaluate the cost and efficacy of the developed AI by applying it to multiple hospitals in Hue, Vietnam, and Da Nang, Vietnam.

It is one of few academic studies on the development and application of AI in LMICs. The study is a product of international collaboration, including epidemiologists, global health experts, professional programmers, and local physicians in Vietnam, facilitating questionnaire development, data collection, and AI development.

As this study is a pilot study, there are several limitations. First, we only used data from a single hospital at Da Nang, Vietnam, so additional validation is required before applying it to other regions. Due to the decrease in the number of patients visiting the hospital, a certain proportion of patients were recruited retrospectively via telephone survey. Although we tried to keep data integrity by reviewing EMR, data validity of retrospective data might have affected the result. Finally, our AI could not diagnose infectious diseases without definite clinical manifestation, such as sepsis. To correctly identify complex diseases and syndromes, we should include in-depth assessments of symptoms and clinical features in the model. To address these shortcomings, we plan to develop assessment questionnaires further, distribute the AI to multiple collaborating hospitals and healthcare centers in Vietnam and assess the efficacy of the AI in collaborating institutions.

## Conclusion

This study is one of few academic studies on AIs in resource-limited settings. Our results implied that even survey-based questionnaires without laboratory or radiologic tests could be beneficial in screening infectious diseases in LMIC. Additional studies on other collaborating institutions will further develop and validate the current model we have developed and provide epidemiologic evidence on the effectiveness of AI application in a resource-limited setting.

## Data availability statement

The raw data supporting the conclusions of this article will be made available by the authors, without undue reservation.

## Ethics statement

The studies involving human participants were reviewed and approved by the institutional review board of Da Nang Hospital. The patients/participants provided their written informed consent to participate in this study.

## Author contributions

Concept and design: KK, M-kL, and SK. Drafting of the manuscript: KK and SK. Statistical analysis: KK, HL, and BK. Obtained funding: M-kL and HS. Administrative, technical, or material support: M-kL, HS, HL, and SK. Supervision: M-kL and SK. All authors had full access to all the data in the study, takes responsibility for the integrity of the data and the accuracy of the data analysis, acquisition, analysis, interpretation of data, and critical revision of the manuscript for important intellectual content.

## Funding

This study was funded by RIGHT Fund (Investment ID RF-TAA-2021-H01). However, the funding source had no role in designing and conducting the study.

## Conflict of interest

Author HS was employed by company Acryl. Authors HL, BK and HS were employed by company FineHealthcare. The remaining authors declare that the research was conducted in the absence of any commercial or financial relationships that could be construed as a potential conflict of interest.

## Publisher's note

All claims expressed in this article are solely those of the authors and do not necessarily represent those of their affiliated organizations, or those of the publisher, the editors and the reviewers. Any product that may be evaluated in this article, or claim that may be made by its manufacturer, is not guaranteed or endorsed by the publisher.
