# Supplementary material for: Development and application of survey-based artificial intelligence for clinical decision support in managing infectious diseases: A pilot study on a hospital in central Vietnam
Source: Front Public Health. 2022 Nov 2;10:1023098. doi: 10.3389/fpubh.2022.1023098 (PMC9683382; doi:10.3389/fpubh.2022.1023098)
Supplement: Supplementary file 1 [file Table_1.DOCX]

**Supplemental Material 1. Classification of disease entity by ICD-10 diagnostic codes**

| **Category** | **ICD-10 Code** | **Disease entity** |
| --- | --- | --- |
| 1. Mosquito-borne diseases | A90 | Dengue fever |
| 1. Mosquito-borne diseases | A91 | Dengue hemorrhagic fever |
| 1. Mosquito-borne diseases | A92.0 | Chikungunya hemorrhagic fever |
| 1. Mosquito-borne diseases | A92.1 | O'nyong-nyong fever |
| 1. Mosquito-borne diseases | A92.2 | Venezuelan equine fever |
| 1. Mosquito-borne diseases | A92.3 | West Nile fever |
| 1. Mosquito-borne diseases | A92.4 | Rift Valley fever |
| 1. Mosquito-borne diseases | A92.8 | Other specified mosquito-borne viral fevers |
| 1. Mosquito-borne diseases | A92.9 | Mosquito-borne viral fever, unspecified |
| 2. Acute infectious gastroenteritis | A00.0 | Cholera due to Vibrio cholerae 01, biovar cholerae |
| 2. Acute infectious gastroenteritis | A00.1 | Cholera due to Vibrio cholerae 01, biovar eltor |
| 2. Acute infectious gastroenteritis | A01.0 | Typhoid fever |
| 2. Acute infectious gastroenteritis | A01.1 | Paratyphoid fever A |
| 2. Acute infectious gastroenteritis | A01.2 | Paratyphoid fever B |
| 2. Acute infectious gastroenteritis | A01.3 | Paratyphoid fever C |
| 2. Acute infectious gastroenteritis | A01.4 | Paratyphoid fever, unspecified |
| 2. Acute infectious gastroenteritis | A02.0 | Samonella enteritis |
| 2. Acute infectious gastroenteritis | A03.0 | Shigellosis due to Shigella dysenteriae |
| 2. Acute infectious gastroenteritis | A03.1 | Shigellosis due to Shigella flexneri |
| 2. Acute infectious gastroenteritis | A03.2 | Shigellosis due to Shigella boydii |
| 2. Acute infectious gastroenteritis | A03.3 | Shigellosis due to Shigella soneei |
| 2. Acute infectious gastroenteritis | A03.8 | Other shigellosis |
| 2. Acute infectious gastroenteritis | A03.9 | Shigellosis, unspecified |
| 2. Acute infectious gastroenteritis | A04.0 | Enteropathogenic Escherichia coli infection |
| 2. Acute infectious gastroenteritis | A04.1 | Enterotoxigenic Escherichia coli infection |
| 2. Acute infectious gastroenteritis | A04.2 | Enteroinvasive Escherichia coli infection |
| 2. Acute infectious gastroenteritis | A04.3 | Enterohaemorrhagic Escherichia coli infection |
| 2. Acute infectious gastroenteritis | A04.4 | Other intestinal Escherichia coli infection |
| 2. Acute infectious gastroenteritis | A04.5 | Campylobacter enteritis |
| 2. Acute infectious gastroenteritis | A04.6 | Enteritis due to Yersinia enterocolitica |
| 2. Acute infectious gastroenteritis | A04.7 | Enterocolitis due to Clostridium difficile |
| 2. Acute infectious gastroenteritis | A04.8 | Other specified bacterial intestinal infections |
| 2. Acute infectious gastroenteritis | A04.9 | Bacerial enteritis NOS |
| 2. Acute infectious gastroenteritis | A05.0 | Foodborne staphylococcal intoxication |
| 2. Acute infectious gastroenteritis | A05.1 | Botulism |
| 2. Acute infectious gastroenteritis | A05.2 | Foodborne Clostridium perfringens [Clostridium welchii] intoxication |
| 2. Acute infectious gastroenteritis | A05.3 | Foodborne Vibrio parahaemolyticus intoxication |
| 2. Acute infectious gastroenteritis | A05.4 | Foodborne Bacillus cereus intoxication |
| 2. Acute infectious gastroenteritis | A05.8 | Other specified bacterial foodborne intoxications |
| 2. Acute infectious gastroenteritis | A05.9 | Bacterial foodborne intoxication, unspecified |
| 2. Acute infectious gastroenteritis | A06.0 | Acute amoebic dysentery |
| 2. Acute infectious gastroenteritis | A06.1 | Chronic intestinal amoebiasis |
| 2. Acute infectious gastroenteritis | A06.2 | Amoebic nondysenteric colitis |
| 2. Acute infectious gastroenteritis | A06.3 | Amoeboma of intestine |
| 2. Acute infectious gastroenteritis | A07.0 | Balantidiasis |
| 2. Acute infectious gastroenteritis | A07.1 | Giardiasis |
| 2. Acute infectious gastroenteritis | A07.2 | Cryptosporidiosis |
| 2. Acute infectious gastroenteritis | A07.3 | Isosporiasis |
| 2. Acute infectious gastroenteritis | A07.8 | Other specified protozoal intestinal diseaess |
| 2. Acute infectious gastroenteritis | A07.9 | Protozoal intestinal disease, unspecified |
| 2. Acute infectious gastroenteritis | A08.0 | Rotaviral enteritis |
| 2. Acute infectious gastroenteritis | A08.1 | Acute gastroenteropathy due to Norovirus |
| 2. Acute infectious gastroenteritis | A08.2 | Adenoviral enteritis |
| 2. Acute infectious gastroenteritis | A08.3 | Other viral entieritis |
| 2. Acute infectious gastroenteritis | A08.4 | Viral intestinal infection, unspecified |
| 2. Acute infectious gastroenteritis | A08.5 | Other specified intestinal infections |
| 2. Acute infectious gastroenteritis | A09 | Other gastroenteritis and colitis of infectious and unspecified origin |
| 3. Respiratory tract infection | A06.5 | Amoebic liver abscess |
| 3. Respiratory tract infection | J00 | Acute nasopharyngitis |
| 3. Respiratory tract infection | J02 | Acute pharyngitis |
| 3. Respiratory tract infection | J02.0 | Streptcoccal pharyingitis |
| 3. Respiratory tract infection | J02.8 | Acute pharyngitis due to other specified organisms |
| 3. Respiratory tract infection | J02.9 | Acute pharyngitis, unspecified |
| 3. Respiratory tract infection | J04.0 | Acute laryngitis |
| 3. Respiratory tract infection | J04.1 | Acute tracheitis |
| 3. Respiratory tract infection | J04.2 | Acute laryngotracheitis |
| 3. Respiratory tract infection | J05.0 | Acute obstructive laryngitis[croup] |
| 3. Respiratory tract infection | J05.1 | Acute epiglottitis |
| 3. Respiratory tract infection | J06.0 | Acute laryngopharyngitis |
| 3. Respiratory tract infection | J06.8 | Other acute upper respiratory infections of multiple sites |
| 3. Respiratory tract infection | J06.9 | Acute upper respiratory infection, unspecified |
| 3. Respiratory tract infection | J09 | Influenza due to certain identified influenza virus |
| 3. Respiratory tract infection | J10.0 | Influenza with pneumonia, seasonal influenza virus identified |
| 3. Respiratory tract infection | J10.1 | Influenza with other respiratory manifestations, seasonal influenza virus identified |
| 3. Respiratory tract infection | J10.8 | Influenza with other manifestations, seasonal influenza virus identified |
| 3. Respiratory tract infection | J11.0 | Influenza with pneumonia, virus not identified |
| 3. Respiratory tract infection | J11.1 | Influenza with other respiratory manifestations, virus not identified |
| 3. Respiratory tract infection | J11.8 | Influenza with other manifestations, virus not identified |
| 3. Respiratory tract infection | J12.0 | Adenoviral pneumonia |
| 3. Respiratory tract infection | J12.1 | Respiratory syncytial virus pneumonia |
| 3. Respiratory tract infection | J12.2 | Parainfluenza virus pneumonia |
| 3. Respiratory tract infection | J12.3 | Human metapneumovirus pneumonia |
| 3. Respiratory tract infection | J12.8 | Other viral pneumonia |
| 3. Respiratory tract infection | J12.9 | Viral pneumonia, unspecified |
| 3. Respiratory tract infection | J13 | Pneumonia due to Streptococcus pneumoniae |
| 3. Respiratory tract infection | J14 | Pneumonia due to Haemophilus influenzae |
| 3. Respiratory tract infection | J15.0 | Pneumonia due to Klebsiella pneumoniae |
| 3. Respiratory tract infection | J15.1 | Pneumonia due to Pseudomonas |
| 3. Respiratory tract infection | J15.2 | Pneumonia due to staphylococcus |
| 3. Respiratory tract infection | J15.3 | Pneumonia due to streptococcus, group B |
| 3. Respiratory tract infection | J15.4 | Pneumonia due to other streptococci |
| 3. Respiratory tract infection | J15.5 | Pneumonia due to Escherichia coli |
| 3. Respiratory tract infection | J15.6 | Pneumonia due to other Gram-negative bacteria |
| 3. Respiratory tract infection | J15.7 | Pneumonia due to Mycoplasma pneumoniae |
| 3. Respiratory tract infection | J15.8 | Other bacterial pneumonia |
| 3. Respiratory tract infection | J15.9 | Bacterial pneumonia, unspecified |
| 3. Respiratory tract infection | J16.0 | Chlamydial pneumonia |
| 3. Respiratory tract infection | J16.8 | Pneumonia due to other specified infectious organisms |
| 3. Respiratory tract infection | J17.0 | Pneumonia in diseases classified elsewhere |
| 3. Respiratory tract infection | J17.1 | Pneumonia in viral diseases classified elsewhere |
| 3. Respiratory tract infection | J17.2 | Pneumonia in mycoses |
| 3. Respiratory tract infection | J17.3 | Pneumonia in parasitic diseases |
| 3. Respiratory tract infection | J17.8 | Pneumonia in other diseases classified elsewhere |
| 3. Respiratory tract infection | J18 | Pneumonia, organism unspecified |
| 3. Respiratory tract infection | J18.9 | Pneumonia, unspecified |
| 3. Respiratory tract infection | J20.0 | Acute bronchitis due to Mycoplasma pneumoniae |
| 3. Respiratory tract infection | J20.1 | Acute bronchitis due to Haemophilus influenzae |
| 3. Respiratory tract infection | J20.2 | Acute bronchitis due to streptococcus |
| 3. Respiratory tract infection | J20.3 | Acute bronchitis due to coxsackievirus |
| 3. Respiratory tract infection | J20.4 | Acute bronchitis due to parainfluenza virus |
| 3. Respiratory tract infection | J20.5 | Acute bronchitis due to respiratory syncytial virus |
| 3. Respiratory tract infection | J20.6 | Acute bronchitis due to rhinovirus |
| 3. Respiratory tract infection | J20.7 | Acute bronchitis due to echovirus |
| 3. Respiratory tract infection | J20.8 | Acute bronchitis due to other specified organisms |
| 3. Respiratory tract infection | J20.9 | Acute bronchitis, unspecified |
| 3. Respiratory tract infection | J21.0 | Acute bronchiolitis due to respiratory syncytial virus |
| 3. Respiratory tract infection | J21.1 | Acute bronchiolitis due to human metapneumovirus |
| 3. Respiratory tract infection | J21.8 | Acute bronchiolitis due to other specified organisms |
| 3. Respiratory tract infection | J21.9 | Acute bronchiolitis, unspecified |
| 3. Respiratory tract infection | J22 | Unspecified acute lower respiratory tract infection |
| 3. Respiratory tract infection | U07.1 | Coronavirus disease, virus identified [COVID-19, virus identified] |
| 3. Respiratory tract infection | U07.2 | Coronavirus disease 2019, virus not identified [COVID-19, virus not identified] |
| 4. Tuberculosis | A15 | Respiratory tuberculosis, bacteriologically and histologically confirmed |
| 4. Tuberculosis | A16 | Respiratory tuberculosis, not confirmed bacteriologically or histologically |
| 5. Sepsis | A02.1 | Salmonella sepsis |
| 5. Sepsis | A20.7 | Septicemic plague |
| 5. Sepsis | A22.7 | Anthrax sepsis |
| 5. Sepsis | A26.7 | Erysipelothrix sepsis |
| 5. Sepsis | A32.7 | Listerial sepsis |
| 5. Sepsis | A40 | Streptcoccal sepsis |
| 5. Sepsis | A41 | Other sepsis |
| 5. Sepsis | A41.51 | Sepsis dueto Pseudomonas |
| 5. Sepsis | A42.7 | Actinomycotic sepsis |
| 5. Sepsis | B37.7 | Candidal sepsis |
| 5. Sepsis | O75.3 | Other infection during labor |
| 5. Sepsis | O85 | Puerperal sepsis |
| 5. Sepsis | P36 | Bacterial sepsis of newborn |
| 5. Sepsis | R65 | Systemic Inflammatory Response Syndrome |
| 6. CNS infection | A06.6 | Amoebic brain abscess |
| 6. CNS infection | A17.0 | Tuberculous meningitis |
| 6. CNS infection | A17.0 | Tuberculous meningitis |
| 6. CNS infection | A17.1 | Meningeal tuberculoma |
| 6. CNS infection | A17.8 | Other tuberculosis of nervous system |
| 6. CNS infection | A17.9 | Tuberculosis of nervous system, unspecified |
| 6. CNS infection | A32.1 | Listerial meningitis and meningoencephalitis |
| 6. CNS infection | A39 | Meningococcal infection |
| 6. CNS infection | A39.0 | Meningococcal meningitis |
| 6. CNS infection | A50.4 | Congenital syphilis |
| 6. CNS infection | A51.4 | Secondary syphilis |
| 6. CNS infection | A52.1 | Symptomatic neurosyphilis |
| 6. CNS infection | A54.8 | Gonococcal meningitis |
| 6. CNS infection | A87.0 | Enteroviral meningitis |
| 6. CNS infection | A87.1 | Adenoviral meningitis |
| 6. CNS infection | B00.3 | Herpesviral meningitis |
| 6. CNS infection | B01.0 | Varicella meningitis |
| 6. CNS infection | B02.1 | Zoster meningitis |
| 6. CNS infection | B05.1 | Measles complicated bymeningitis |
| 6. CNS infection | B06.0 | Rubella with neurological complications |
| 6. CNS infection | B26.1 | Mumps meningitis |
| 6. CNS infection | B37.5 | Candidal meningitis |
| 6. CNS infection | B38.4 | Coccidioidomycosis meningitis |
| 6. CNS infection | B45.1 | Cerebral cryptococcosis |
| 6. CNS infection | G00.0 | Haemophilus meningitis |
| 6. CNS infection | G00.1 | Pneumococcal meningitis |
| 6. CNS infection | G00.2 | Streptococcal meningitis |
| 6. CNS infection | G00.3 | Staphylococcal meningitis |
| 6. CNS infection | G00.8 | Other bacterial meningitis |
| 6. CNS infection | G00.9 | Bacterial meningitis, unspecified |
| 6. CNS infection | G01 | Meningitis in bacterial diseases classified elsewhere |
| 6. CNS infection | G02.0 | Meningitis in viral diseases classified elsewhere |
| 6. CNS infection | G02.1 | Meningitis in mycoses |
| 6. CNS infection | G02.8 | Meningitis in other specified infectious and parasitic diseases classified elsewhere |
| 6. CNS infection | G03 | Meningitis due to other and unspecified causes |
| 6. CNS infection | G04.0 | Acute disseminated encephalitis |
| 6. CNS infection | G04.1 | Human T-cell lymphotropic virus associated myelopathy |
| 6. CNS infection | G04.2 | Bacterial meningoencephalitis and meningomyelitis, NEC |
| 6. CNS infection | G04.8 | Other encephalitis, myelitis and encephalomyelitis |
| 6. CNS infection | G04.9 | Encephalitis, myelitis and encephalomyelitis, unspecified |
| 6. CNS infection | G05.0 | Encephalitis, myelitis and encephalomyelitis in bacterial diseases classified elsewhere |
| 6. CNS infection | G05.1 | Encephalitis, myelitis and encephalomyelitis in viral diseases classified elsewhere |
| 6. CNS infection | A85.1 | Adenoviral encephalitis |
| 6. CNS infection | B25.8 | Other cytomegaloviral disease |
| 6. CNS infection | A85.0 | Enteroviral encephalitis |
| 6. CNS infection | B00.4 | Herpesviral encephalitis |
| 6. CNS infection | B05.0 | Measles complicated by encephalitis |
| 6. CNS infection | B26.2 | Mumps encephalitis |
| 6. CNS infection | B01.1 | Varicella encephalitis |
| 6. CNS infection | B02.0 | Zoster encephalitis |
| 6. CNS infection | B06.0 | Encephalitis, myelitis and encephalomyelitis in other infectious and parasitic diseases classified elsewhere |
| 6. CNS infection | B57.4 | Chagas’ disease (chronic) with nervous system involvement |
| 6. CNS infection | B60.2 | Naegleriasis |
| 6. CNS infection | G06.0 | Intracranial abscess and granuloma |
| 6. CNS infection | G06.1 | Intraspinal abscess and granuloma |
| 6. CNS infection | G06.2 | Extradural and subdural abscess, unspecified |
| 6. CNS infection | G07 | Intracranial and intraspinal abscess and granuloma in diseases classified elsewhere |
| 7. Viral hepatitis | B15 | Acute hepatitis A |
| 7. Viral hepatitis | B16 | Acute hepatitis B |
| 7. Viral hepatitis | B17.0 | Acute delta-(super)infection in chronic hepatitis B |
| 7. Viral hepatitis | B17.1 | Acute hepatitis C |
| 7. Viral hepatitis | B17.2 | Acute hepatitis E |
| 7. Viral hepatitis | B17.8 | Other specified acute viral hepatitis |
| 7. Viral hepatitis | B17.9 | Acute viral hepatitis, NOS |
| 7. Viral hepatitis | B18 | Chronic viral hepatitis |
| 7. Viral hepatitis | B19 | Unspecified viral hepatitis |
| 7. Viral hepatitis | B15.0 | Hepatitis A with hepatic coma |
| 7. Viral hepatitis | B15.9 | Hepatitis A without hepatic coma |
| 7. Viral hepatitis | B16.0 | Acute hepatitis B with delta-agent (coinfection) with hepatic coma |
| 7. Viral hepatitis | B16.1 | Acute hepatitis B with delta-agent (coinfection) without hepatic coma |
| 7. Viral hepatitis | B16.2 | Acute hepatitis B without delta-agent with hepatic coma |
| 7. Viral hepatitis | B16.9 | Acute hepatitis B without delta-agent and without hepatic coma |
| 7. Viral hepatitis | B17.0 | Acute delta-(super)infection of hepatitis B carrier |
| 7. Viral hepatitis | B17.1 | Acute hepatitis C |
| 7. Viral hepatitis | B17.2 | Acute hepatitis E |
| 7. Viral hepatitis | B17.8 | Other specified acute viral hepatitis |
| 7. Viral hepatitis | B17.9 | Acute viral hepatitis, unspecified |
| 7. Viral hepatitis | B18.0 | Chronic viral hepatitis B with delta-agent |
| 7. Viral hepatitis | B18.1 | Chronic viral hepatitis B without delta-agent |
| 7. Viral hepatitis | B18.2 | Chronic viral hepatitis C |
| 7. Viral hepatitis | B18.8 | Other chronic viral hepatitis |
| 7. Viral hepatitis | B18.9 | Chronic viral hepatitis, unspecified |
| 7. Viral hepatitis | B19.0 | Unspecified viral hepatitis with hepatic coma |
| 7. Viral hepatitis | B19.9 | Unspecified viral hepatitis without hepatic coma |
