# Supplementary material for: Development and application of survey-based artificial intelligence for clinical decision support in managing infectious diseases: A pilot study on a hospital in central Vietnam
Source: Front Public Health. 2022 Nov 2;10:1023098. doi: 10.3389/fpubh.2022.1023098 (PMC9683382; doi:10.3389/fpubh.2022.1023098)
Supplement: Supplementary file 2 [file Table_2.DOCX]

**Supplemental Material 2. Survey questionnaire used for data collection and artificial intelligence development.**

**Part 1. Demographic characteristics**

**A1. When is your birthday?** ⬜⬜⬜⬜ / ⬜⬜ / ⬜⬜

**A2. What is your sex?** 1) Male 2) Female

**A3. What is your occupation?**

1) Legislators, senior officials, executives and managers.

2) Professionals

3) Associate professionals and technicians

4) Clerical support workers

5) Service workers and sales workers.

6) Skilled agricultural, forestry and fishery workers

7) Craft and related trades workers

8) Plant and machine operators and assemblers

9) Elementary occupations

10) Armed forces officers

98) Retired or unemployed (including housewife)

99) Not elsewhere classified (e.g., freelance workers)

**A4. To which degree have you received formal education?**

1) Elementary school level or lower (<6 years)

2) Middle school level (7-9 years)

3) High school level (10-12 years)

4) College level or above

**A5. What is your address?**

**A6. Do you own your house?** 1) No 2) Yes

**A7. With how many people do you share your household? It doesn’t have to be a family member.** ⬜⬜ people

**A8. Which of these describes your personal income per month? It can apply VND or USD**

1) 0 - 2 million VND 2) 2-4 million VND 3) 4 - 6million VND

4) 6 - 8 million VND 5) 8-10 million VND 6) >10 million VND

**A9. Which of the following is the main water source you obtain drinking water from?**

1) Tap water 2) Bottled water 3) Wells 4) Rivers, lakes or ponds

5) Groundwater 6) Rainwater 7) Others

**A10. Which type of lavatory facility does your household have?**

1) Flush toilet 2) Squat toilet 3) Pit latrine 4) Chamber pot 5) Others

**Part 2.** Body measurements and medical history

**B1. Height:** ⬜⬜⬜ **cm**

**B2. Body weight:** ⬜⬜.⬜ **kg**

- **BMI:** ⬜⬜.⬜⬜ (automatically calculated after inputting B1 & B2)

**B3. Waist circumference:** ⬜⬜⬜.⬜**cm**

**B4. Vital signs**

B4-1. Systolic body pressure: ⬜⬜⬜mmHg

B4-2. Diastolic body pressure: ⬜⬜⬜mmHg

B4-3. Pulse rate: ⬜⬜⬜ beats/min

B4-4. Respiratory rate: ⬜⬜ /min

**B5. Have you ever been diagnosed of following diseases by doctor during lifetime?**

| **Diseases** | **History of diagnosis** | | |
| --- | --- | --- | --- |
|  | **No** | **Yes (Age at diagnosis)** | **N/A** |
| Hypertension | 1) | 2) (______) | 9) |
| Hyperlipidemia or dyslipidemia | 1) | 2) (______) | 9) |
| Diabetes mellitus | 1) | 2) (______) | 9) |
| Cardiovascular and cerebrovascular diseases | 1) | 2) (______) | 9) |
| Thyroid diseases | 1) | 2) (______) | 9) |
| Depressive disorders | 1) | 2) (______) | 9) |
| Liver cirrhosis | 1) | 2) (______) | 9) |
| Malignant neoplasms _(1)_ _________________ | 1) | 2) (______) | 9) |
| Malignant neoplasms _(2)_ _________________ | 1) | 2) (______) | 9) |

**B6. Have your parents, siblings and children ever been diagnosed of following diseases by doctor?**

| **Diseases** | **History of diagnosis** | | |
| --- | --- | --- | --- |
|  | **No** | **Yes (Age at diagnosis)** | **N/A** |
| Hypertension | 1) | 2) (______) | 9) |
| Hyperlipidemia or dyslipidemia | 1) | 2) (______) | 9) |
| Diabetes mellitus | 1) | 2) (______) | 9) |
| Cardiovascular and cerebrovascular diseases | 1) | 2) (______) | 9) |
| Thyroid diseases | 1) | 2) (______) | 9) |
| Depressive disorders | 1) | 2) (______) | 9) |
| Liver cirrhosis | 1) | 2) (______) | 9) |
| Malignant neoplasms _(1)_ ____________________ | 1) | 2) (______) | 9) |
| Malignant neoplasms _(2)_ ____________________ | 1) | 2) (______) | 9) |

**Part 3. Current symptoms**

**※ Please check all symptoms you have and describe when these symptoms first developed.**

**C1. General weakness**

**<Systematic>**

C1-1 General weakness: 1) No 2) Yes, started in ⬜⬜ days

C1-2 Fever: 1) No 2) Yes, started in ⬜⬜ days

C1-3 Chill: 1) No 2) Yes, started in ⬜⬜ days

C1-4 Fatigue: 1) No 2) Yes, started in ⬜⬜ days

C1-5 Loss of appetite: 1) No 2) Yes, started in ⬜⬜ days

C1-6 Change in body weight: 1) No 2) Yes, started in ⬜⬜ days

C1-7 Pain: 1) No 2) Yes, started in ⬜⬜ days

**<HEENT>**

C2-1 Headache: 1) No 2) Yes, started in ⬜⬜ days

C2-2 Neck stiffness: 1) No 2) Yes, started in ⬜⬜ days

C2-3 Dizziness: 1) No 2) Yes, started in ⬜⬜ days

C2-4 Vertigo: 1) No 2) Yes, started in ⬜⬜ days

C2-5 Sore throat: 1) No 2) Yes, started in ⬜⬜ days

C2-6 Rhinorrhea: 1) No 2) Yes, started in ⬜⬜ days

C2-7 Nasal stiffness: 1) No 2) Yes, started in ⬜⬜ days

C2-8 Nasal bleeding: 1) No 2) Yes, started in ⬜⬜ days

**<Respiratory>**

C3-1 Cough: 1) No 2) Yes, started in ⬜⬜ days

C3-2 Sneeze: 1) No 2) Yes, started in ⬜⬜ days

C3-3 Sputum: 1) No 2) Yes, started in ⬜⬜ days

C3-4 Hemoptysis: 1) No 2) Yes, started in ⬜⬜ days

C3-5 Dyspnea: 1) No 2) Yes, started in ⬜⬜ days

C3-6 Dyspnea on exertion (DOE): 1) No 2) Yes, started in ⬜⬜ days

C3-7 Orthopnea: 1) No 2) Yes, started in ⬜⬜ days

**<Cardiovascular>**

C4-1 Chest discomfort or pain (angina): 1) No 2) Yes, started in ⬜⬜ days

C4-2 Radiating pain: 1) No 2) Yes, started in ⬜⬜ days

C4-3 Palpitation: 1) No 2) Yes, started in ⬜⬜ days

C4-4 Coldness in limbs: 1) No 2) Yes, started in ⬜⬜ days

C4-5 Cyanosis: 1) No 2) Yes, started in ⬜⬜ days

**<Gastrointestinal>**

C5-1 Anorexia: 1) No 2) Yes, started in ⬜⬜ days

C5-2 Nausea: 1) No 2) Yes, started in ⬜⬜ days

C5-3 Vomiting: 1) No 2) Yes, started in ⬜⬜ days

C5-4 Diarrhea: 1) No 2) Yes, started in ⬜⬜ days

C5-5 Constipation: 1) No 2) Yes, started in ⬜⬜ days

C5-6 Abdominal pain: 1) No 2) Yes, started in ⬜⬜ days

C5-6-1 Site of pain: 1) RUQ 2) RLQ 3) LUQ 4) LLQ


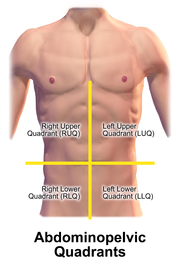


C5-7 Hematemesis: 1) No 2) Yes, started in ⬜⬜ days

C5-8 Melena: 1) No 2) Yes, started in ⬜⬜ days

C5-9 Hematochezia: 1) No 2) Yes, started in ⬜⬜ days

C5-10 Jaundice: 1) No 2) Yes, started in ⬜⬜ days

C5-11 Ascites: 1) No 2) Yes, started in ⬜⬜ days

**<Genitourinary>**

C6-1 Dysuria: 1) No 2) Yes, started in ⬜⬜ days

C6-2 Gross hematuria: 1) No 2) Yes, started in ⬜⬜ days

C6-3 Urgency: 1) No 2) Yes, started in ⬜⬜ days

C6-4 Frequency: 1) No 2) Yes, started in ⬜⬜ days

C6-5 Hesitancy: 1) No 2) Yes, started in ⬜⬜ days

C6-6 Flank pain: 1) No 2) Yes, started in ⬜⬜ days

C6-7 Edema: 1) No 2) Yes, started in ⬜⬜ days

**<Skin & Musculoskeletal>**

C7-1 Myalgia: 1) No 2) Yes, started in ⬜⬜ days

C7-2 Joint pain: 1) No 2) Yes, started in ⬜⬜ days

C7-3 Joint stiffness: 1) No 2) Yes, started in ⬜⬜ days

C7-4 Rash: 1) No 2) Yes, started in ⬜⬜ days

C7-5 Heat: 1) No 2) Yes, started in ⬜⬜ days

**<Neurologic>**

C8-1 Altered consciousness: 1) No 2) Yes, started in ⬜⬜ days

C8-2 Altered orientation: 1) No 2) Yes, started in ⬜⬜ days

C8-3 Altered cognition: 1) No 2) Yes, started in ⬜⬜ days

C8-4 Altered sensory function: 1) No 2) Yes, started in ⬜⬜ days

C8-5 Altered motor function: 1) No 2) Yes, started in ⬜⬜ days

C8-6 Tremor: 1) No 2) Yes, started in ⬜⬜ days

C8-7 Spasm: 1) No 2) Yes, started in ⬜⬜ days

C8-8 Plegia: 1) No 2) Yes, started in ⬜⬜ days

C8-9 Paresis: 1) No 2) Yes, started in ⬜⬜ days

C8-10 Gait disturbance: 1) No 2) Yes, started in ⬜⬜ days

C8-11 Aphasia: 1) No 2) Yes, started in ⬜⬜ days

C9. Did you take any medications after the symptom onset? If so, name medications you took.

1) No 2) Yes, medication 1 , medication 2 , medication 3

**Part 4. Infectious disease history**

D1. Did you contact someone with respiratory symptoms (cough, sputum, rhinorrhea, hemoptysis) within 14 days?

1) No 2) Yes

D2. Did you contact someone who is a confirmed COVID-19 confirmed patient or a close contactor to COVID-19 patients?

1) No 2) Yes

D3. Did you wear masks when going out or visiting public places? 1) No 2) Yes

D4. Did your family members or roommates had similar symptoms listed above? If so, who has it and when did their symptom start? 1) No 2) Yes

D5. Did you contact someone who is a confirmed tuberculosis patient?
1) No 2) Yes

D6. Did you travel within 14 days? If so, where was your destination?

1) No 2) Yes, destination 1 , destination 2

D7. Did you eat raw/uncooked/less cooked/spoiled fish, clam, shellfish, shrimps or other seafoods? If so, when did you consume it?

1) No 2) Yes, ⬜⬜⬜ hours ago

D8. Did you eat raw/uncooked/less cooked/spoiled meat, egg, milk, butter or other dairy products? If so, when did you consume it?

1) No 2) Yes, ⬜⬜⬜ hours ago

D9. Did you eat other types of raw/uncooked/less cooked/spoiled foods? If so, when did you consume it?

1) No 2) Yes, ⬜⬜⬜ hours ago

D10. (If one or more answers for D7-D9 is “Yes”)

Did people who you shared foods with have been ill as well?

1) No 2) Yes

D11. Have you been to lakes, ponds, rivers, marshlands or seashores within 2 weeks?

1) No 2) Yes

D12. Did you have a direct contact with soil within 72 hours?

1) No 2) Yes

D13. Did you get injured by rusted iron (nails, needles, etc.)?

1) No 2) Yes

D14. Have you been to forests, grasslands or rainforests within 2 weeks?

1) No 2) Yes

D15. Have you been bitten by mosquitos within 2 weeks?

1) No 2) Yes

D16. Have you been bitten by tick within 2 weeks?

1) No 2) Yes

D17. Have you been bitten by other insects within 2 weeks?

1) No 2) Yes

D18. Did you have direct contact with animals within 2 weeks? If so, which animals did you have contact with?

1) No 2) Yes,

D19. Have you been bitten by animals within 2 weeks, If so, which animal bit you?

1) No 2) Yes,
