# Supplementary material for: Development and application of survey-based artificial intelligence for clinical decision support in managing infectious diseases: A pilot study on a hospital in central Vietnam
Source: Front Public Health. 2022 Nov 2;10:1023098. doi: 10.3389/fpubh.2022.1023098 (PMC9683382; doi:10.3389/fpubh.2022.1023098)
Supplement: Supplementary file 3 [file Table_3.DOCX]

**Supplemental Material 3. Optimized value of hyperparameters**

| **Hyperparameter** | **Value** |
| --- | --- |
| Learning rate | 0.01 |
| Max_depth | 9 |
| N_estimator | 10000 |
| Early_stopping_rounds | 2000 |
| Eval_metric | Accuracy |
