# Supplementary material for: Development and application of survey-based artificial intelligence for clinical decision support in managing infectious diseases: A pilot study on a hospital in central Vietnam
Source: Front Public Health. 2022 Nov 2;10:1023098. doi: 10.3389/fpubh.2022.1023098 (PMC9683382; doi:10.3389/fpubh.2022.1023098)

Supplemental Material 5 Feature importance of variables included in artificial intelligence model by disease entity.

(1) Mosquito-borne disease

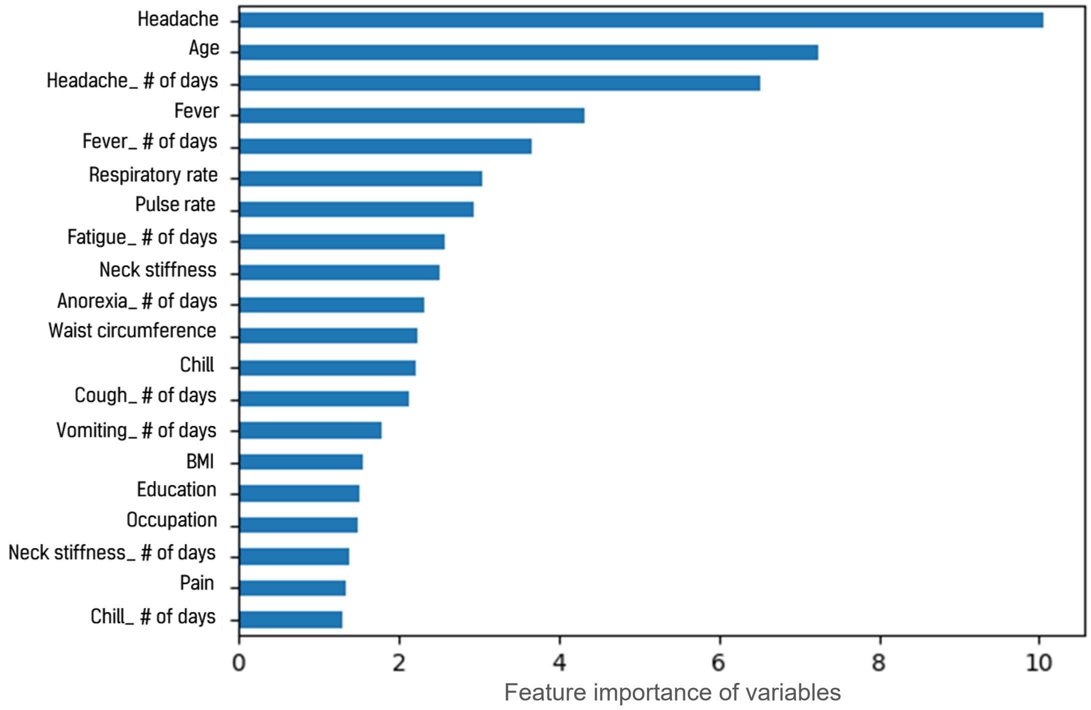

(2) Acute gastroenteritis

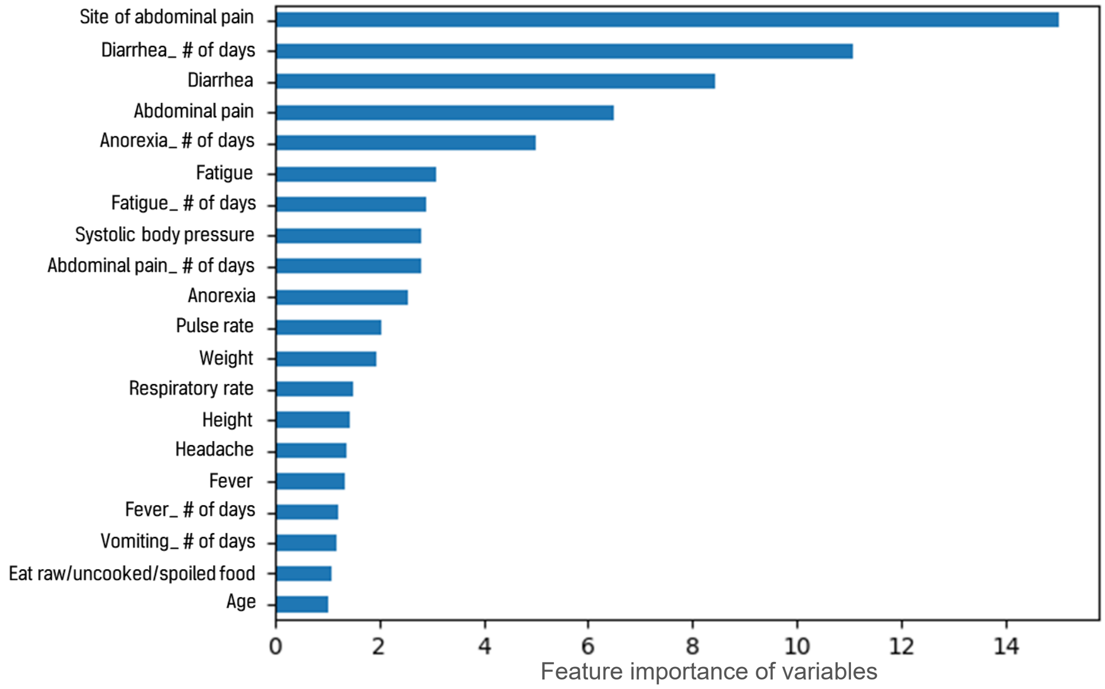

(3) Respiratory tract infection including COVID-19

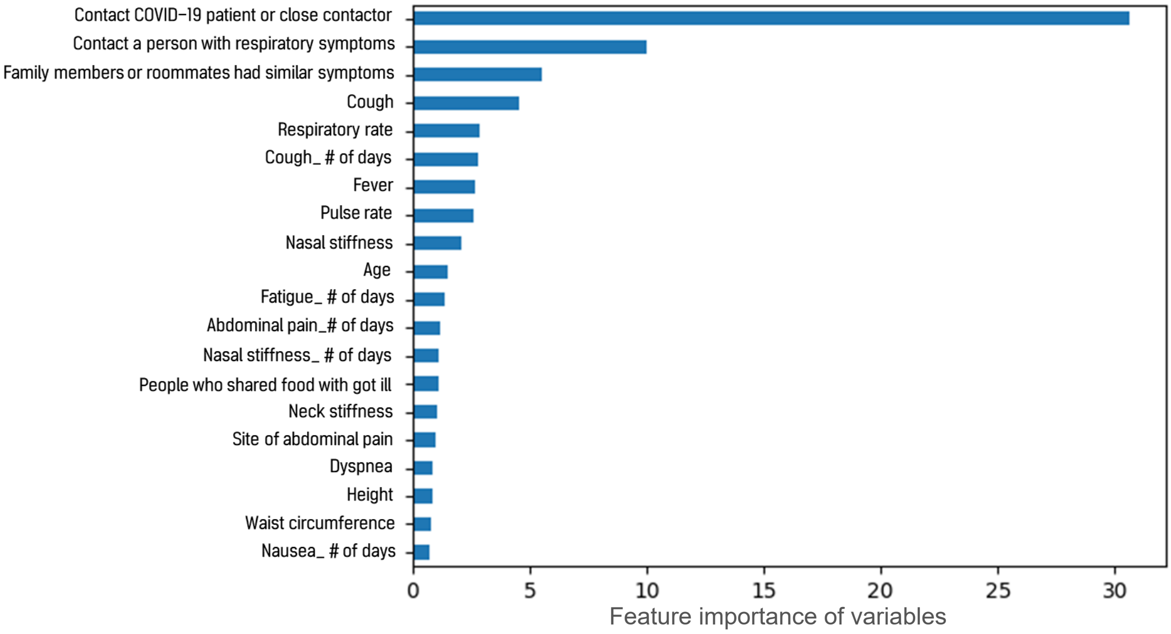

(4) Pulmonary tuberculosis

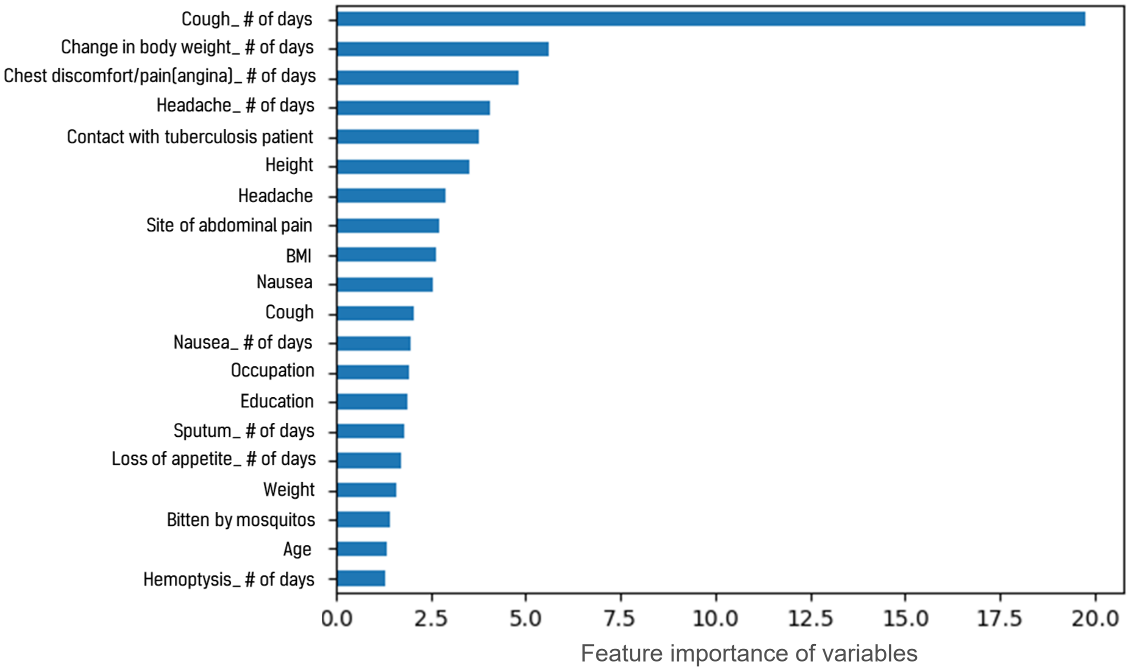

(5) Sepsis

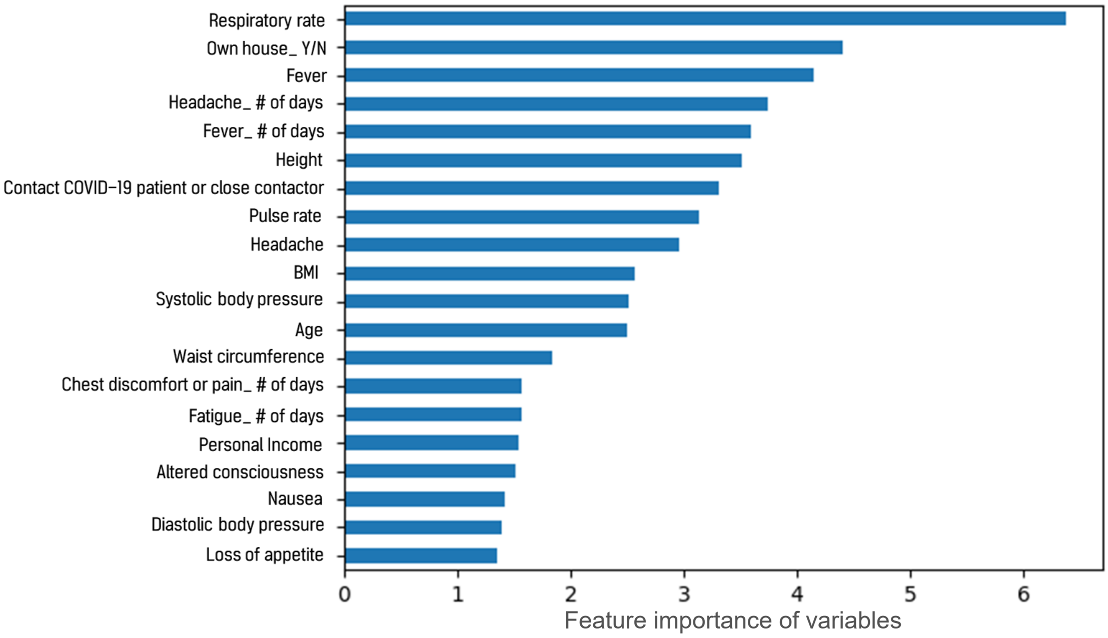

(6) Central nervous system infection

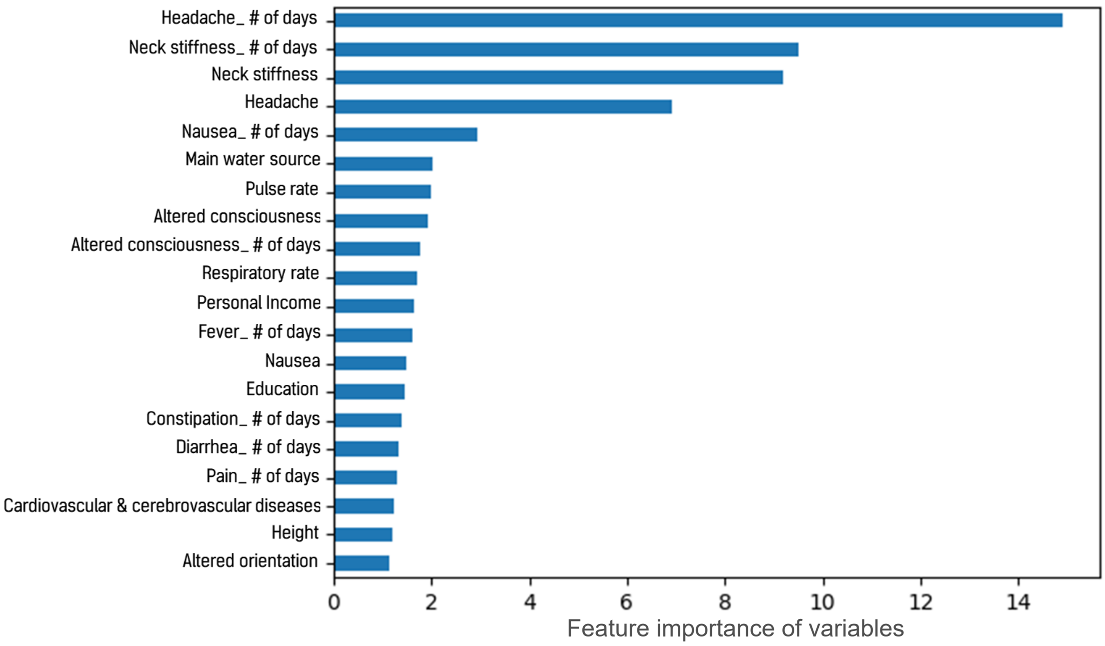

(7) Viral hepatitis

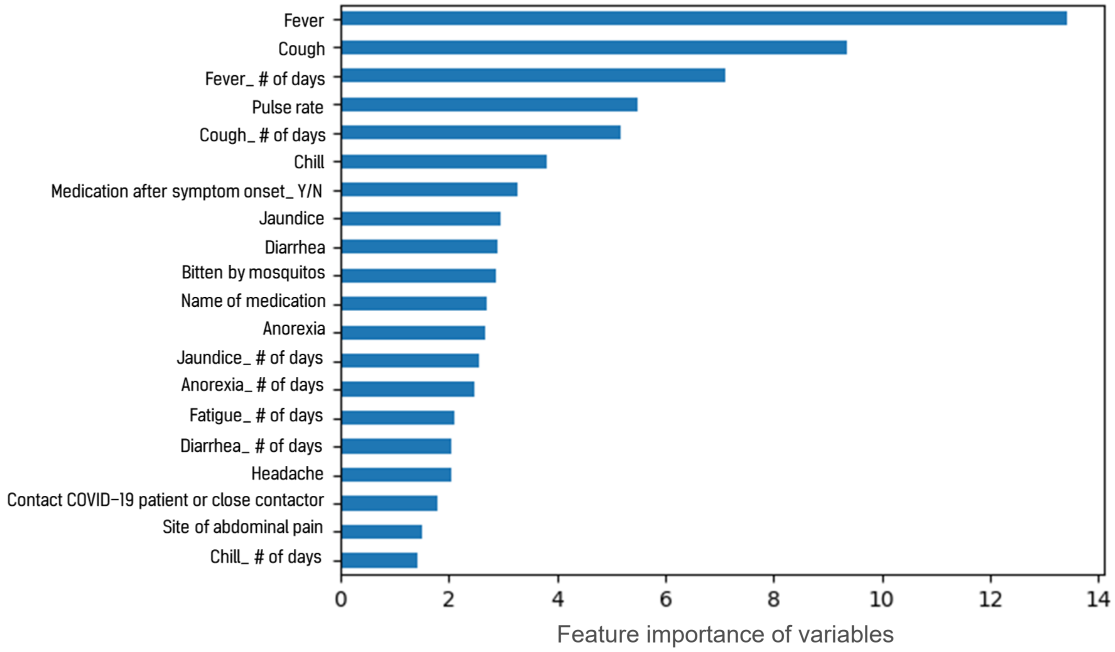

Supplement: Supplementary file 4 [file Image_2.PDF]
